# Mortuary Statistics and Meteorological Report

**Published:** 1880-07

**Authors:** 


### JUNE REPORT OF DEATHS AND INTERMENTS IN BALTIMORE

| CAUSE OF DEATH. | No. | CAUSE OF DEATH. | No. | CAUSE OF DEATH. | No |
|---|---|---|---|---|---|
| Abscess, Bowels | 1 | Dropsy (Gen'l) | 3 | Jaundice | 2 |
| " Chest | 1 | Drowned | 10 | Myelitis | 2 |
| Anæmia | 2 | Dysentery | 25 | Mal-formation | 1 |
| Abscess of Liver | 1 | Entero colitis | 8 | Malnutrition | 16 |
| Angina Pectoris | 1 | Erysipelas | 1 | Marasmus | 31 |
| Albuminuria | 2 | Embolism | 1 | Measles | 1 |
| Apoplexy | 7 | Fever Catarrh | 2 | Meningitis | 13 |
| Ascites | 1 | " Intermittent | 2 | " Tubercular | 17 |
| Asthma | 4 | " Puerperal | 1 | Neurasthenia | 1 |
| Asthenia | 7 | " Gastric | 1 | Necrosis of Pelvis | 1 |
| Atrophy | 1 | " Remittent | 4 | Neuritis | 1 |
| Bright's Disease | 7 | " Scarlet | 29 | Old Age | 15 |
| Burns | 1 | " Typhoid | 13 | Osteo Sarcomb of Sarcrum. | 1 |
| Capillary Bronchitis | 2 | " Typhus | 1 | Paralysis | 15 |
| Carbuncle | 1 | " Malarial | 1 | Pneumonia | 20 |
| Cancer, Breast | 2 | " Typho malarial | 1 | " Typhoid | 1 |
| " Bone | 1 | Fracture Temporal Bone | 1 | " Pleuro | 3 |
| " Liver | 1 | " Thigh | 1 | " broncho | 1 |
| " Larynx | 1 | Gangrene Angina | 1 | Poison | 1 |
| " Neck | 2 | " Throat | 1 | Premature Birth | 17 |
| " Hip | 1 | Gastro Enteritis | 8 | Protracted Labor | 1 |
| " Peritoneum | 1 | Hernia, Strangulated | 1 | Puerpural scarlatina | 1 |
| " Uterus | 4 | " Umbilical | 3 | " mania | 1 |
| " Cervical Gland | 1 | Hemorrhage of Bowels | | Pyæmia | 1 |
| " Stomach | 3 | " Brain | | Rheumatism | 3 |
| Casualties | 1 | " Lungs | | Rickets | 1 |
| Child Birth | 4 | " Uterus | | Smoking a strong cigar | 1 |
| Cholera Infantum | 188 | Hæmatitis | 1 | Scrofula | 2 |
| " Morbus | 6 | Hemorrhage Umbilical | 3 | Softening of Brain | 3 |
| Cirrhosis of Liver | 1 | Hydrocephalus | 7 | Suppression of menses | 1 |
| Caries of Sternum | 1 | Indigestion | 2 | Suicide | 2 |
| Concussion of Brain | 1 | Inanition | 14 | Syphilis | 4 |
| Congestion of Brain | 11 | Icterus | 1 | Tabes mesenterica | 4 |
| " Lungs | 5 | Insanity | 1 | Tetanus | 4 |
| Consumption of Lungs | 87 | Imperfect Circulation | 1 | Trismus Nascentium | 2 |
| Convulsions | 32 | Inflammation of Brain | 2 | Tumor, Leg | 1 |
| Croup | 6 | " Bowels | 9 | " Abdomen | 1 |
| Cyanosis | 2 | " Stomach. | 6 | " Ovarian | 1 |
| Dentition | 13 | " Bronchi | 4 | Ulcer Bowels | 1 |
| Delirium Tremens | 1 | " Kidneys | 1 | " Stomach | 1 |
| Dia rhœa | 21 | " Larynx | 1 | Unknown Adult | 2 |
| Diphtheria | 13 | " Liver | 1 | " Infantile | 1 |
| Disease of Heart | 23 | " Pericardium | 1 | Whooping Cough | 13 |
| " Liver | 1 | " Peritoneum | 2 | Wounds of Spine | 1 |
| " Messentery Gland. | 1 | " Pleura | 3 | | |
| " Spine | 1 | Intussusception | 1 | | 869 |
| " Stomach | 1 | Intemperance | 1 | | |

THE DEATHS REGISTERED IN THE MONTH INCLUDE.

| | | | |
|---|---|---|---|
| Under 1 year | 403 | Between 20 and 30 " | 36 |
| Between 1 and 2 years | 71 | " 30 and 40 " | 47 |
| " 2 and 5 years | 44 | " 40 and 50 " | 37 |
| | | " 50 and 60 " | 53 |
| Under 5 years | 518 | " 60 and 70 " | 36 |
| Between 5 and 10 years | 53 | " 70 and 80 " | 46 |
| " 10 and 15 " | 10 | " 80 and 90 " | 11 |
| " 15 and 20 " | 21 | " 90 and 100 " | 1 |

NATIVITY, ETC.

| | |
|---|---|
| United States, White | 559 |
| Colored | 204 |
| Foreign | 106 |
| | 869 |
| Still Birth | 60 |

Among the decedents were 61 above the age of 70 years, whose combined ages were 4,704 years, averaging 77 years, 0 months.

Estimated Population, 393,796. White, 338,384. Colored, 55,412. Annual Death Rate during the month, whole population, per 1,000—26.46. White, 23,60. Colored, 44.50.

By Order: A. R. CARTER, Secretary.

JAMES A. STEUART, M. D., Commissioner of Health and Registrar.

U. S. SIGNAL OFFICE, BALTIMORE, MD., July 1, 1880.

### METEOROLOGICAL SUMMARY FOR THE MONTH OF JUNE, 1880.

### PRESSURE.

Mean Monthly Barometer - - - - - 30.019 inches.
" " " - - - for past 10 years 29,994 inches.
Highest Barometer - - - - - - 30,306 inches, on the 19th.
Lowest " - - - - - - - - 29,679 inches, on the 13th.
Monthly Range of Barometer - - - - 0,627 inches.

### TEMPERATURE.

Mean Monthly Thermometer - - - - 73.3 degrees.
" " " - - for past 10 years 74.2 degrees.
Highest Thermometer - - 95 degrees on 12th & 24th. Monthly Range - - 43 degrees.
Lowest " - - - 52 " " 3d. Mean Daily Range - 17.5 "

### WIND, ETC.

Prevailing Direction of Wind, West.
Mean Velocity of Wind, 6.3 miles per hour.
Highest Velocity of Wind, 26 " "

Total Amount of Rainfall, 5.48 inches.
Number of Days on which Rain fell, 14.
Average rainfall for June, 3.30 inches.

ROBERT SEYBOTH, in charge.

